# Moles, Warts and Birth-marks

**Published:** 1880-12

**Authors:** 


### MOLES, WARTS AND BIRTH-MARKS.

We could never quite understand why any gentleman, and particularly any lady, should consent to remain conspicuous, by reason of any ugly mole on the face, when the defect may be easily and safely remedied.

We have extirpated a "hat full"—"be the same more or less"—and have done our work so thoroughly, that there is not the ghost of a chance that a single mole will return to torment its former possessor. Our plan is this: Where there is but one mole, not too large, we simply freeze it with a spray of ether, and then make a curved incision in the direction of the folds of the skin, from a quarter to half an inch in length, on each side of the mole, and close to it, so as to close it between the curved incisions; and then we remove the mole with the small portion of skin on either side of it. We then sponge the wound till it stops bleeding, and draw the edges of skin accurately together with several very narrow strips of court plaster. In three or four days it will heal; generally so that not the least scar or line can be seen. If there are two or more moles to be taken out, or one large one, we take our patients to a neighboring dentist who administers gas, and while under the influence, we can dispose of three or four moles; and then apply the plasters afterward. Thus the whole operation is painless, and entirely safe.

Acids and caustics should never be used to destroy moles. They always leave scars, which are often greater deformities than the moles themselves. Beside, the operation is tedious and painful. Warts can generally be driven away in a few days, by wetting a piece of salammoniac and rubbing the warts with it two or three times a day.

The treatment of birth-marks depends so much upon their size and character that it would be impossible to give instructions that would apply to all cases. If small, and located where the skin is loose, they may be removed at one operation, or a portion may be removed and the wound allowed to heal, and then another portion removed. This gives the skin time to adapt itself to the position before occupied by the birth-mark, and when properly done leaves no noticeable scar.

Any educated physician would soon become expert in these operations of minor surgery.

